# Performance analysis of PID controller and fuzzy logic controller for DC-DC boost converter

**DOI:** 10.1371/journal.pone.0281122

**Published:** 2023-10-19

**Authors:** Amil Daraz, Abdul Basit, Guoqiang Zhang

**Affiliations:** 1 College of Information Science and Electronic Engineering, Zhejiang University, Hangzhou, China; 2 School of Information Science and Engineering, NingboTech University, Ningbo, China; J.C. Bose University of Science and Technology, YMCA, INDIA, INDIA

## Abstract

Demand of Power electronics converter is increased due to the rapid increase of green re-sources. The distributed nature of uncontrolled renewable energy resources are connected with the grid via power electronics converter. DC-DC converters are high-frequency current conversion circuits that use transformers, inductors, and capacitors to smooth switching noise into regulated DC voltages. In this study, a comparative performance analysis of Proportional integral Derivative (PID) and fuzzy logic controller (FLC) for DC-DC Boost Converter is presented. PID-based controller for dynamic boosting of DC-DC boost converter was replaced by Fuzzy Inference system (FIS) to study the system behavior. Five different membership functions were performed to observe the response of the system for DC -DC boost converter with FIS controller. The performances of FIS-based controller and PID-based controller for DC-DC boost converter were compared in detail. Also, the proposed techniques for the switching mode of DC-DC converter have been applied. The performance analysis of both PID and fuzzy controller was simulated using MAT LAB. The results of FIS based approach are better than PID based controller in terms of transient responses.

## Introduction

Demand of Power electronics converter is increased due to the rapid increase of green re-sources. The distributed nature of uncontrolled renewable energy resources are connected with the grid via power electronics converter [1]. Depending on the input voltage, a DC/DC converter will either decrease or increase the output dc voltage. If the output voltage can be stepped up, a boost converter must be used. If the voltage requirement is step-down, then a buck converter must be used. When both types of converters (step-up and step-down) are needed to cover the load, a buck-boost converter is used. For stabilizing a certain input dc voltage, DC-DC converters are essentially used with the required output voltage value. Therefore, for different voltage levels, different variants of DC/DC converters are employed [2, 3].

The new three-port converter that merge Buck-Boost and dual-active bridge converters has been suggested by author in reference [4] and concludes that flexible step-up or step-up and byway power flows can be achieved. To demonstrate that the Buck-Boost converter could handle an exceptionally wide range of voltage at the energy storage terminal, Tahri et al. [5] proposed a non-inverting Buck-Boost converter. Due to this different voltage levels, different DC/DC converters are required for different power requirements. For many years, PI and PID controllers have been used primarily in DC-DC converters. Due to their modesty and less expense, they are broadly used in industrial appliances. However, when applied to non-linear nature objects, PI Controller is unable to adopt and approach the best performance [5, 6]. As a result, voltage regulation of the converters is a challenging task, particularly when the operating range is wide. However, some of the authors also used PID controller for DC-DC boost converter. For In-stance, the researchers in [6] used PID controller for DC-DC boost converter and the efficacy of the suggested approach has been compared with PI controller. The results obtained from the suggested method was better than PI controller at the cost of complexity addition and larger settling time. Moreover, fractional order PID controller has been applied by Huili et al. [7] for DC-CD boost converter. The results yielded from the suggested techniques have been compared with PI controller. The fuzzy logic controller(FLC) approach is completely different because it does not require mathematical modeling or computation and does not become too complicated if simple assumptions are made. In the control field, fuzzy set theory is widely used, with some applications to DC-to-DC converter systems [6, 8–11]. The DC -to- DC converter, also called as switched-mode power supply (SMPS), consists primarily of power semiconductor devices that serve as electronic controls. The commitment cycle was changed utilizing the analysis control loop to keep the yield voltage constant with little consideration of voltage variations and weight variations [12]. The usual nonlinear characteristics of the boost converter were produced by the action of the trading devices [13]. The converters, because of their useless nonlinear phenomena, require a controller with a genuine measure of lively response [14]. The limits of PID controllers are continuously regulated to account for disturbance conditions and changed weights [15]. The disadvantages of the PID include delayed responses to unexpected changes in the stack or tightening of the data voltage. It was primarily the responsibility of the electronic design centers to determine the feasibility of performance changes. Trading difficulties was an important performance measure for pulse width modulation converters [16]. Thus, fuzzy logic and the Brain Framework’s fake-sharp control were quite capable of clearly verifying, flexibly designing, and controlling nonlinear dynamic structures [17]. The nomenclature for different terms is presented in Table 1.

**Table 1:** Nomenclature.

Acronym	Definition	Acronym	Definition
DC	Direct Current	BC	Boost Converter
PI	Proportional Integral	PID	Proportional Integral Derivative
FLC	Fuzzy logic controller	MF	Membership function
FIS	Fuzzy inference system	SMPS	switched-mode power supply
BB	Buck boost	LSF	load side filter
CM	Continuous mood	DCM	Discontinuous mood
ZN	Ziegler- Nichols	PWM	Pulse width modulation
PS	Positive small	NS	Negative small
PB	Positive big	TSK	Takagi sugeno and kang
NB	Negative big	ZO	Zero order
OS	Over shoot	RT	Rise time
Ts	Time settling	RL	Resistive load

Fuzzy logic resembles a human decision methodology for vague and inaccurate information. This makes the real-world problems considerably simpler and is based on truth rather than the usual truth or falseness or 1/0 as Boolean logic. The fuzzy logic approach of control encourages people to learn, without requiring effort, the unpredictability of machines, since it lets them assume the correct points without a clear idea of the path to reach. By utilizing the fuzzy logic control concept, qualitative and heuristic considerations that cannot be handled by conventional control theory can be used for control function in a systematic form. In references [18, 19] FLC are used to handle the non linearity of the system and does not need an exact mathematical structure with vague input. FLC typically lags behind other controllers in terms of complexity, non linearity, or the ability to define a system for which a well-effective knowledge exists. It was proposed as a tool for dealing with ambiguous, unresolved, or qualitative decision making complications. In the intelligent control of complex dynamic systems, controllers that combine perceptive and conventional methods are commonly used [20].

Despite its numerous advantages, FLC is not without its drawbacks. The most significant of these is that when developing a fuzzy controller, the trial and error method is typically required. In order to develop a fuzzy controller, no systematic procedure has been established in [21–24]. Typically, FLC performance is not known until the design phase is complete. As a result, stability analysis is challenging [25–29] to calculate the bandwidth and phase/gain boundary of an FLC. Heuristic knowledge can be incorporated into the controller’s fuzzy logic implementation by taking advantage of the fuzzy logic implementation. This can result in a more effective nonlinear controller that outperforms the linear counterpart that was originally developed. It is possible to make the improvement in such a way that it does not impair the performance or stability of the controller when dealing with small signals. Furthermore, improved large-signal system dynamic performance can be attained by using this technique. One significant advantage of the suggested FLC design method over current approaches is the reduced amount of iterative trial-and-error labor involved in the process overall [30]. Furthermore, before the design process is completed, the proposed system’s small signal performance and stability are understood, saving time and money. The contribution regarding the proposed method are described below.

- In literature fuzzy logic controllers have been applied for the proposed problem, however, the various structure of FLC controller has not been addressed.
- The literature also revealed that said problem with varying voltage levels and loads have not been incorporated properly.
- Heuristic knowledge based rules can be incorporated into the controller’s fuzzy logic implementation.
- Five different membership functions were carried out to observe the response of the system for DC-DC boost converter using FIS controller.
- The parameters of the proposed controllers are optimized by a Ziegler-Nichols (ZN) based closed loop strategy to enhance the stability of the system.
- Further, the proposed techniques have been assessed on different modes of the converter.

## Materials and methods

### Working principle of boost converter

**Fig 1:**
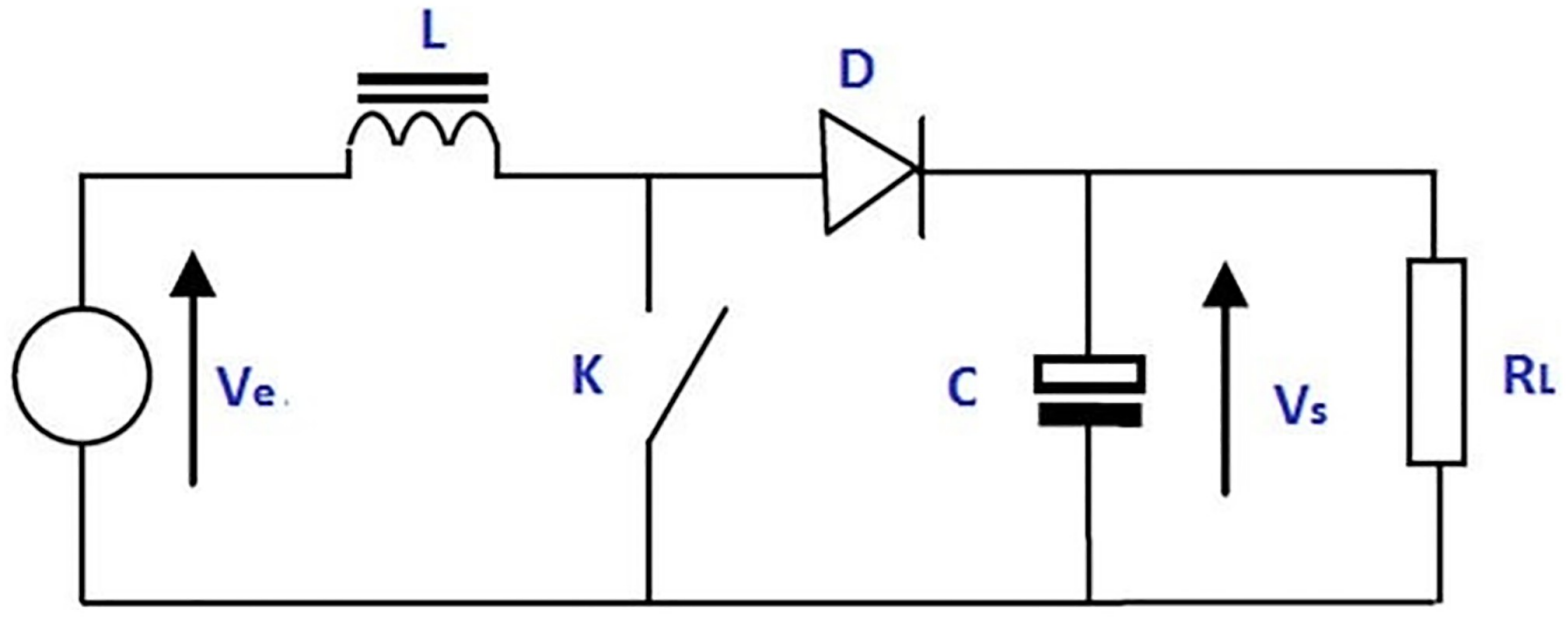
Structure of boost converter.

The boost converter, also called a step-up converter, is a DC-to-DC converter that increases voltage and decreases current from a given input (supply) to the desired output (load). Double semiconductors (transistor and diode) and an energy storage component make up this variety of switched-mode power supply (inductor, capacitor, or both). It is common practice to add capacitor-based filters to the converter’s output (load-side filter) and input to smooth out the voltage (supply-side filter). The basic structure of the boost converter is depicted in Fig 1. The output voltage of a step-up converter is increased relative to the input voltage in the same way that the voltage of a step-up transformer is increased relative to the input voltage. There is no difference between the input and output powers because of the rule of conservation of energy [23].

$$\begin{array}{c} P_{in}=P_{out}. \end{array}$$ (1)

Because Ve is less than Vs in a boost converter, hence the output current is smaller than input current and can be states as:

$$\begin{array}{c} I_{in}>I_{out},V_{e}<V_{s}. \end{array}$$ (2)

The primary driving principle of a boost converter is the inductor’s tendency to produce and reverse polarity when the magnetic field is rapidly fluctuated. When the switch is turned off, current flows in the direction of the winding and a small magnetic field is induced, thereby allowing energy to be accumulated in the inductor. When the switch is opened, a higher impedance will decrease the current. During the on-site conversion, the inductor current will decrease until the current persists to the load. Therefore, the capacitor is charged by the diode (D) from two sources in series which cause higher voltages [18, 31]. There are two modes of boost converter: 1) Continuous type (2) Discontinuous type

1) Continuous mode: For the inductor current to remain constant between cycles, the DC voltage across the inductor must be at zero in the steady state. Because the inductor’s voltage drop is directly proportional to the inductor’s current rise. So average value of Vs is

$$\begin{array}{c} Ve=(1-D)Vo \end{array}$$ (3)

Where Ve represents the input voltage, D represents the duty cycle, and Vs represents the output voltage. The input voltage (Vi) appears across the inductor after switch S is closed, resulting in a time-varying inductor current (IL) over time (t).

$$\begin{array}{c} \frac{\Delta I_{L}}{\Delta t}=\frac{V_{i}}{L} \end{array}$$ (4)

Where L represents the value of inductor. For the ending of On-state, IL is increased and can be represented as:

$$\begin{array}{c} \Delta I_{LOn}=\frac{1}{L}\int_{0}^{t}V_{i}dt=\frac{V_{i}}{L}dt \end{array}$$ (5)

If we consider a diode with no voltage drop and a capacitor large enough to keep its voltage constant and can be represented as:

$$\begin{array}{c} V_{i}-V_{0}=L\frac{d_{IL}}{dt} \end{array}$$ (6)

Variation of IL through the Off-period can be represented as:

$$\begin{array}{c} \Delta I_{LOff}=\int_{T}^{L}\left(V_{i}-V_{s}\right)\frac{dt}{L}=\left(V_{i}-V_{0}\right)\left(1-D\right)\frac{T}{L} \end{array}$$ (7)

Considering that the entire system is in a constant state, the quantity of energy utilized at the start and conclusion of a communication cycle must be identical. The inductor’s stored energy can be expressed as:

$$\begin{array}{c} E=\frac{LI^{2}}{2} \end{array}$$ (8)

Therefore, the current required at the start and at the end of the commutation cycle must be equal and overall, the current has remained constant

$$\begin{array}{c} \Delta I_{ON}+\Delta I_{Off}=0 \end{array}$$ (9)

$$\begin{array}{c} V_{s}\left(1-D\right)=V_{e} \end{array}$$ (10)

So

$$\begin{array}{c} \frac{V_{s}}{V_{e}}=\frac{1}{1-D} \end{array}$$ (11)

2) Discontinuous mode: The inductor current value drops to zero in this mode, which is typical of DC-DC converters. However, in continuous conduction mode, the inductor current is constantly flowing and never stops. For this reason, in discontinuous conduction mode, the inductor current does not remain constant throughout the cycle but rather decays to zero well before the end of the period. The inductance mode of continuous conduction is greater than the inductance mode of discontinuous conduction. If the diode current is greater than the ripple current, the diode will always be turned on, regardless of the polarity of the applied to the switch. When the current through the DC leg of the inductor is less than through the RL, the overall current through the diode is zero. Until the diode is opened, the diode will cease to conduct, and its voltage will be at zero. To generate a higher output voltage, the DCM circuit’s duty cycle ratio is based on both the input and output voltages and the switching frequency. Discontinuous inductance for the boost converter as:

$$\begin{array}{c} LDCM=V_{s}(Vo-Vs)2fVo \end{array}$$ (12)

Duty cycle for DCM is

$$\begin{array}{c} D=\left(V_{o}-V_{s}\right)V_{o}\sqrt{(}E) \end{array}$$ (13)

## Implementation and results

### 0.1 PID based controller for boost converter

**Fig 2:**
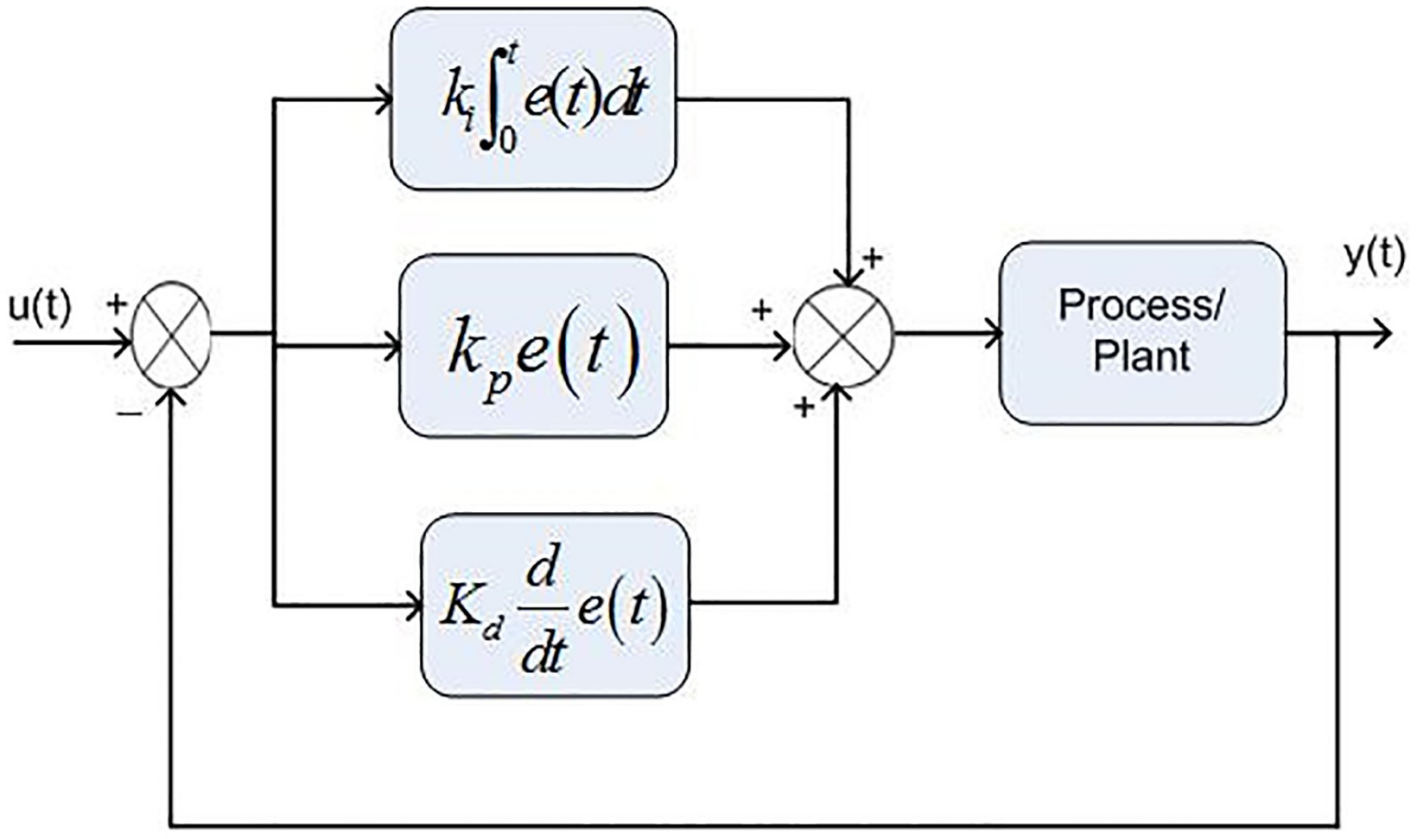
Basic structure of PID controller.

**Fig 3:**
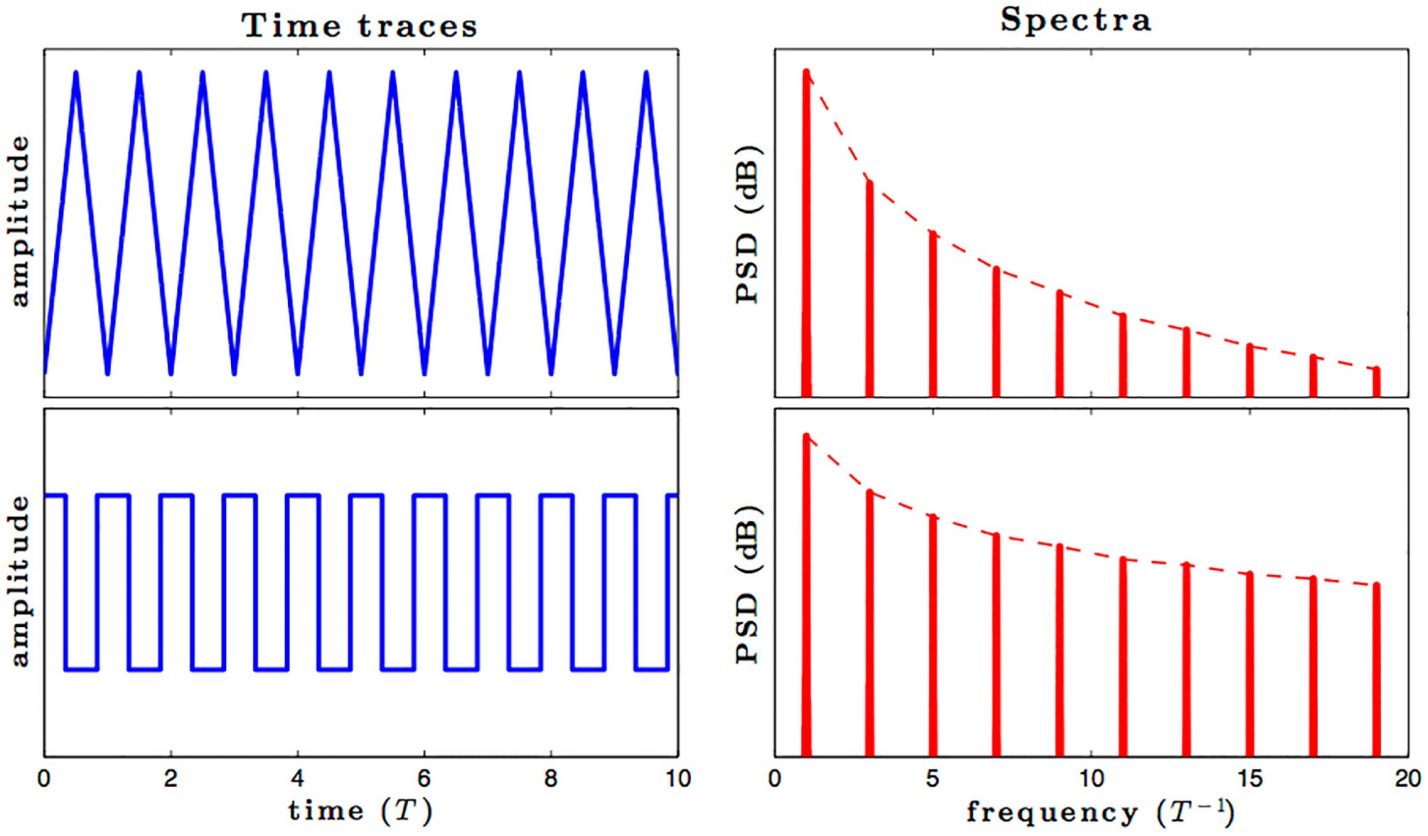
Comparison of PWM signal with a triangular/ saw tooth.

**Fig 4:**
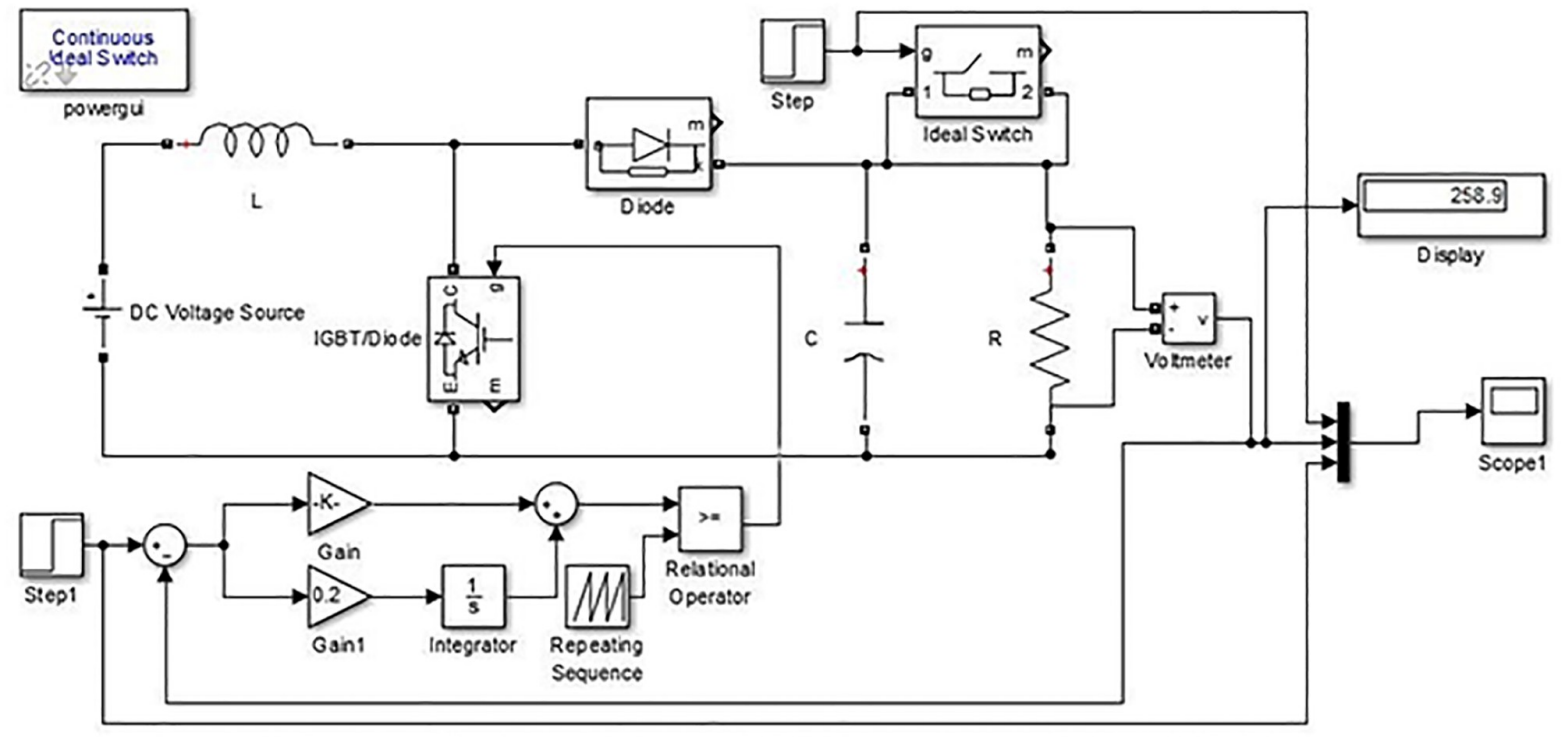
Simulink diagram of a boost converter with PID controller considering case-1, 2 and 3.

**Fig 5:**
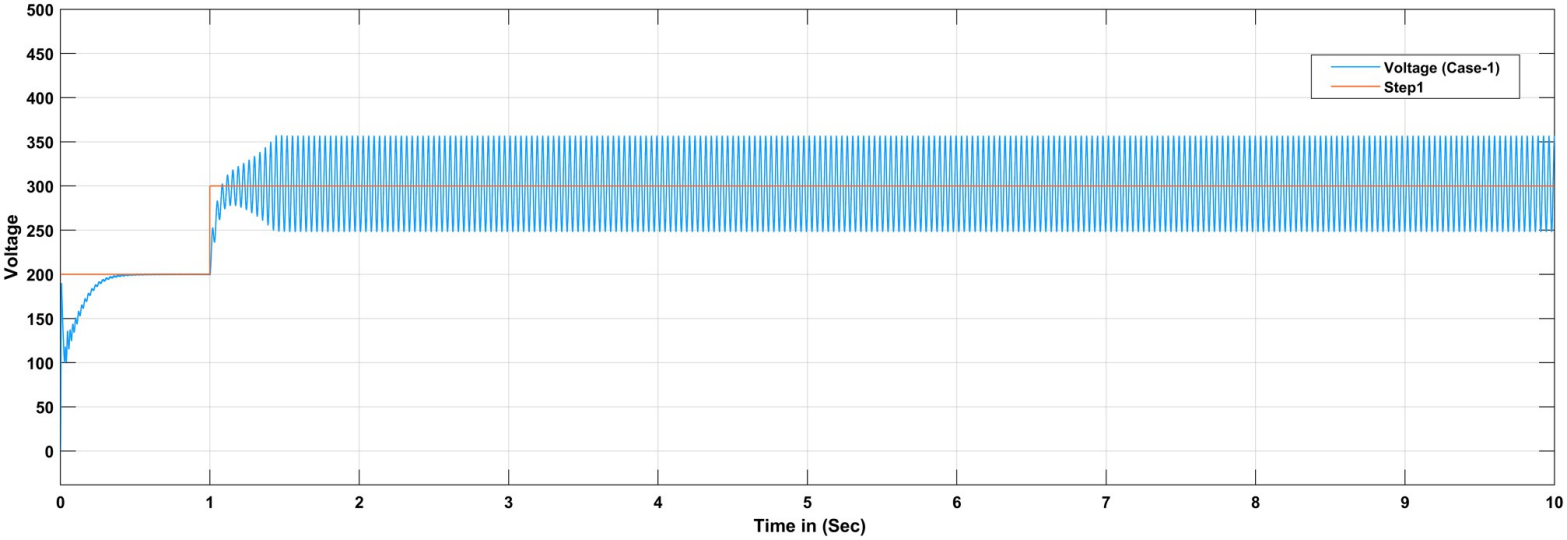
Output response of boost converter with PID controller considering case-2.

**Fig 6:**
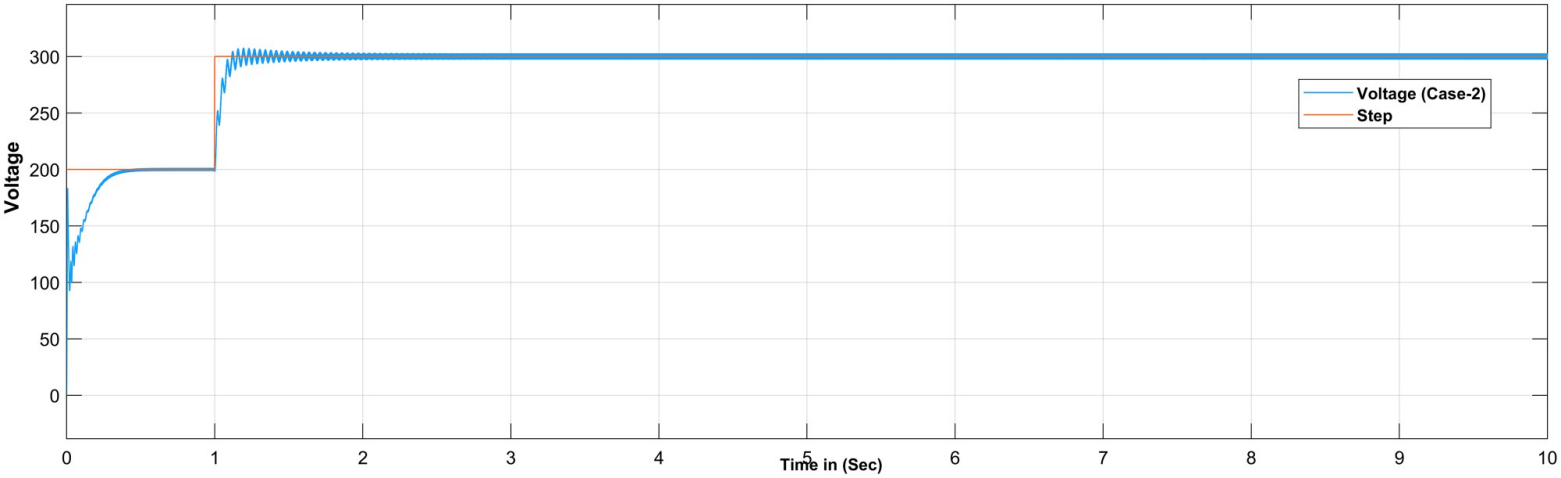
Simulation result of boost converter using PID controller considering case-3.

**Fig 7:**
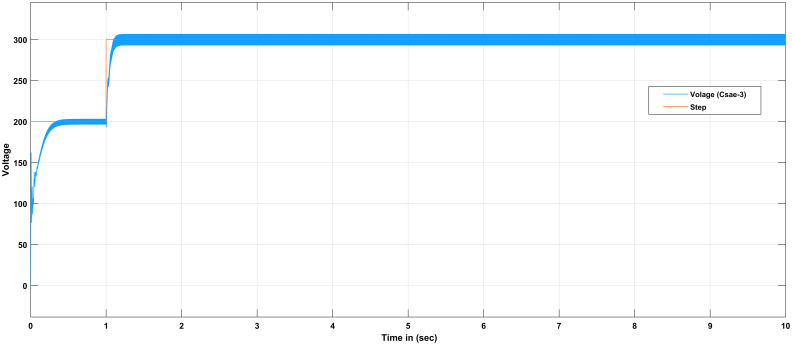
Simulation result of boost converter using PID controller considering case-3.

**Fig 8:**
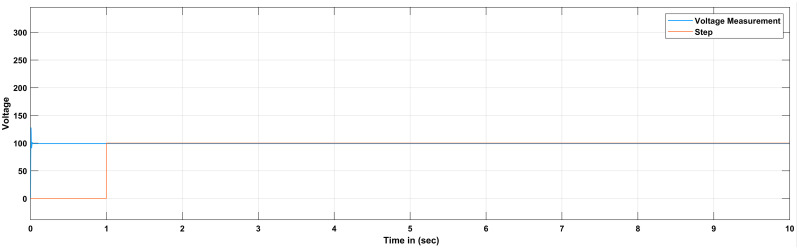
Simulation result of boost converter using PID controller with no load.

The proportional gain in PID controller increased the control signal and overshoot of the system and also it does not eliminate the steady-state error. By including an integral component in the controller, the rising time is decreased, the overshoot and settling times are increased, and the steady-state error is decreased [32]. The response of the suggested system has been assisted for three cases, the reference voltage is increased from an initial value of 200V to 300V after 1 seconds and the resistive load is decrease from 30 ohms to 150 ohms. The basic construction of PID controller is depicted in Fig 2. Closed loop based Ziegler- Nichols (ZN) method is employed to tune the gains of the proposed controller and the values are given in Table 2. The PID gain is determined in this work by comparing the actual boost converter output to a 200–300 V reference voltage that is close to the desired output. The error signal is obtained and then passed through a proportional gain of 0.002 and an integral gain of 0.2. The resulting modulation control signal is then summed with a repeating saw tooth signal which is depicted in Fig 3. Fig 3 shows an example of how PWM signal is compared to a triangular/ saw tooth waveform to generate a required response. Three different scenarios have been validated through the simulink model as defected in Fig 4. In case 1 the simulation was run for 10 seconds using Matlab Simulink tool. Here the reference voltage increases from 200V to 300V after 1 seconds. In case-1 the single resistive load is considered 30 ohms. Fig 5 shows the response of the output obtained for case-1. As the reference voltage increases from 200V to 300V at t = 1 seconds, the ripple also became larger and fluctuated from 260V to 340V. In scenario 2, the simulation was also run for 10 second using Matlab Simulink tool. In this case the resistive load is decreased from 30 ohms to 15 ohms. Fig 6 shows the output response of the PID controller with load resistance R = 15 ohms. While in case 3, the simulation was run for 10 seconds using Matlab Simulink tool. Here in this case two resistor are connected in parallel each resistor have value 30 ohms which make the total resistive load 15 ohms. The output response of the system considering case-3 is shown in Fig 7. The results shown in Fig 7 is the output response for case 3. As the reference voltage increases from 200V to 300V at t = 1 seconds, the ripple also became small and fluctuated from 280 to 320V. Fig 8 represents the response of the system with no load and the transient response parameters are obtained in Table 5. It can be observed from the Fig 8 and also from Table 5 that the system perform very well with no load and quickly stable at 0.02 sec.

**Table 2:** Parameters of the boost converter with PID controllers.

Parameters	Values	Parameters	Values
V*_in_*	100V	Inductor(L)	4mH
Capacitor (C)	100*μ*F	Resistor(R)	30 ohm
Frequency (f)	2KHz	Proportional (K_*p*_)	0.002
Integral (K_*i*_)	0.22	Derivative (K_*d*_)	10

#### 0.1.1 Fuzzy logic control with boost converter

**Fig 9:**
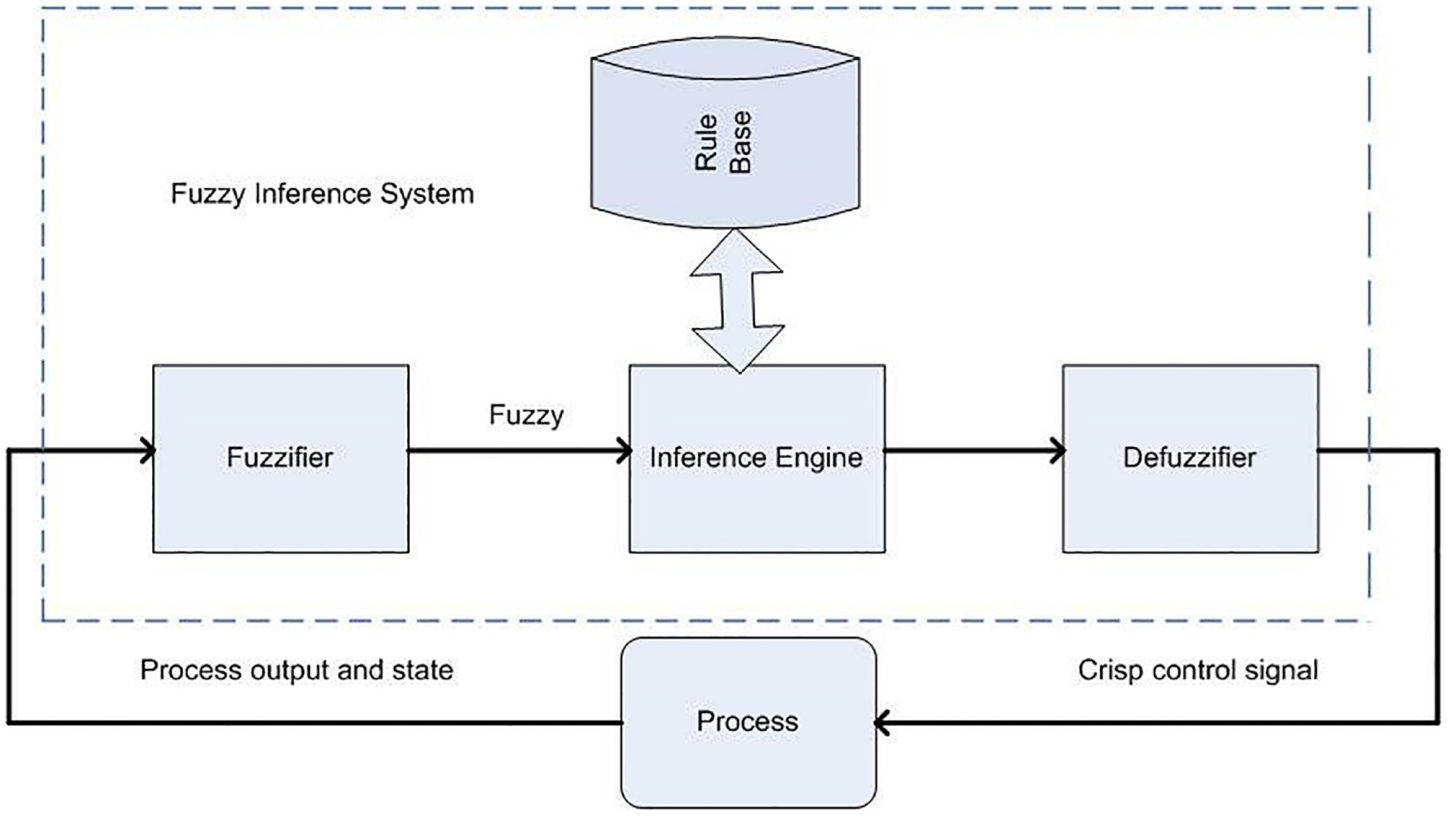
Basic structure of fuzzy inference system (FIS).

**Fig 10:**
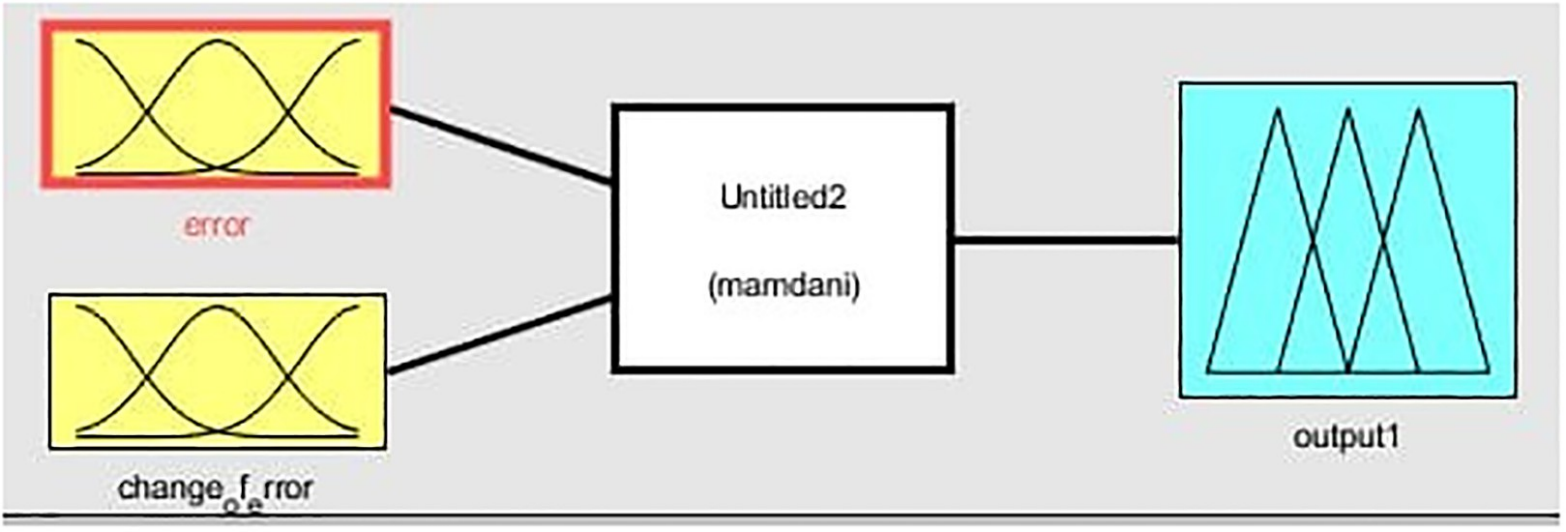
Structure diagram of FLC.

The primary disadvantage of PID controllers is their inability to adapt with nonlinear systems and approach the improved performance. It will have a dynamic response, produce overshoot, have a long settling time, and long rise time, all of which will affect the boost converter’s output voltage (step-up). The basic structure of FIS is given in Fig 9. The process of decomposing a system’s input into one or more fuzzy sets is called fuzzification. There are numerous curve types that can be used for MF, but trapezoidal or triangular-shaped MF are the most frequently used due to their ease of representation in embedded controllers [21–23]. The knowledge base is a collection of rules, while the data base is formed through the conversion of crisp to fuzzy input. Data base defines the MF of the fuzzy set that is used in the fuzzy rule and rule base consist of the principle of IF-THEN. Defuzzification: it converts the fuzzy value obtained from the fuzzy inference engine to a crisp value. There are two main types of fuzzy inference systems to consider when designing a FIS: the mamdani type and the takagi, sugeno, and kang type (TSK). In the case of mamdani FIS, the resulting membership function (MF) is also fuzzy. Since the membership functions in TSK are not fuzzy in nature, the explicability is lacking. Additionally, in the TSK FIS, the subsequent membership functions can have as many parameters as input variables, which results in a higher degree of freedom in the design than in the mamdani FIS, allowing for greater design flexibility. So in this work we used the mamdani FIS. No mathematical model is required for FLC rather than that, they are based on a general understanding of the plant There are two inputs and one output in this fuzzy logic block. Firstly, there is the control error, which is defined as the difference between the reference signal and the output signal, and secondly, there is the change in error. We use a triangular membership function in this study to represent the error, the change in the error, the output variable x and mamdani type FIS. The basis structure of FLC is given in Fig 10.

**Fig 11:**
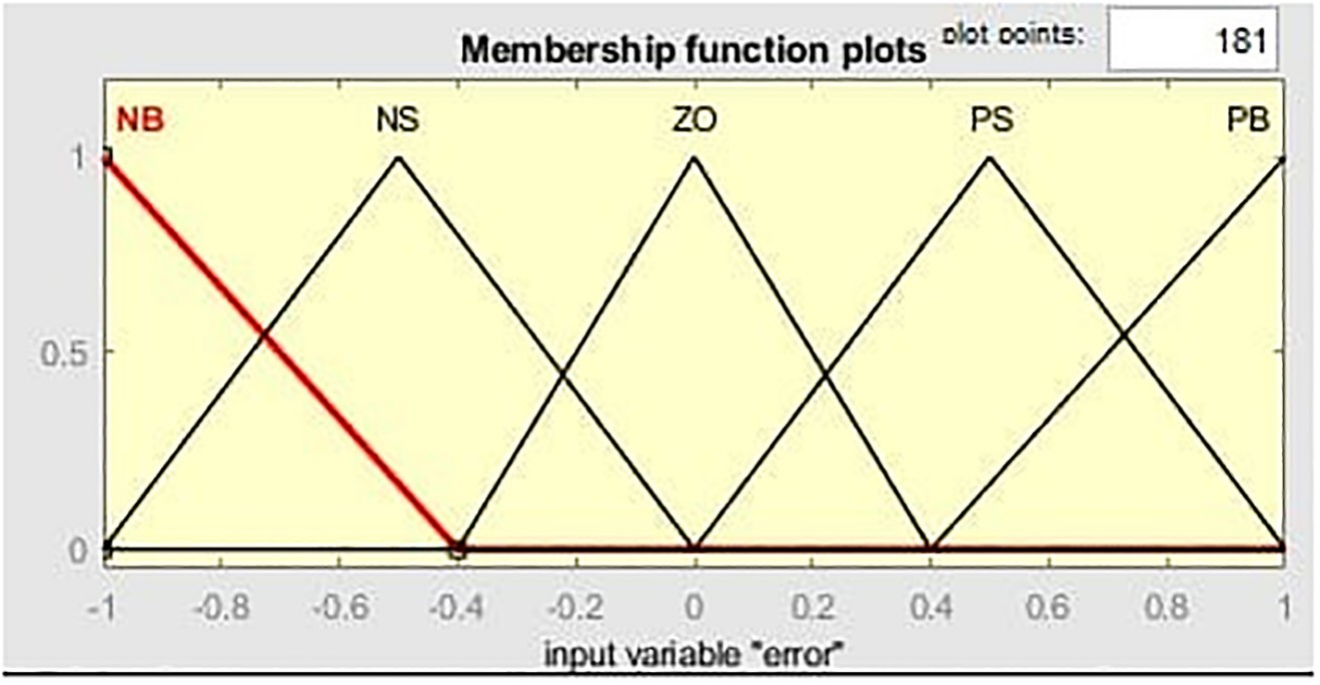
Membership function (MF) for the input linguistic variable ‘error’.

**Fig 12:**
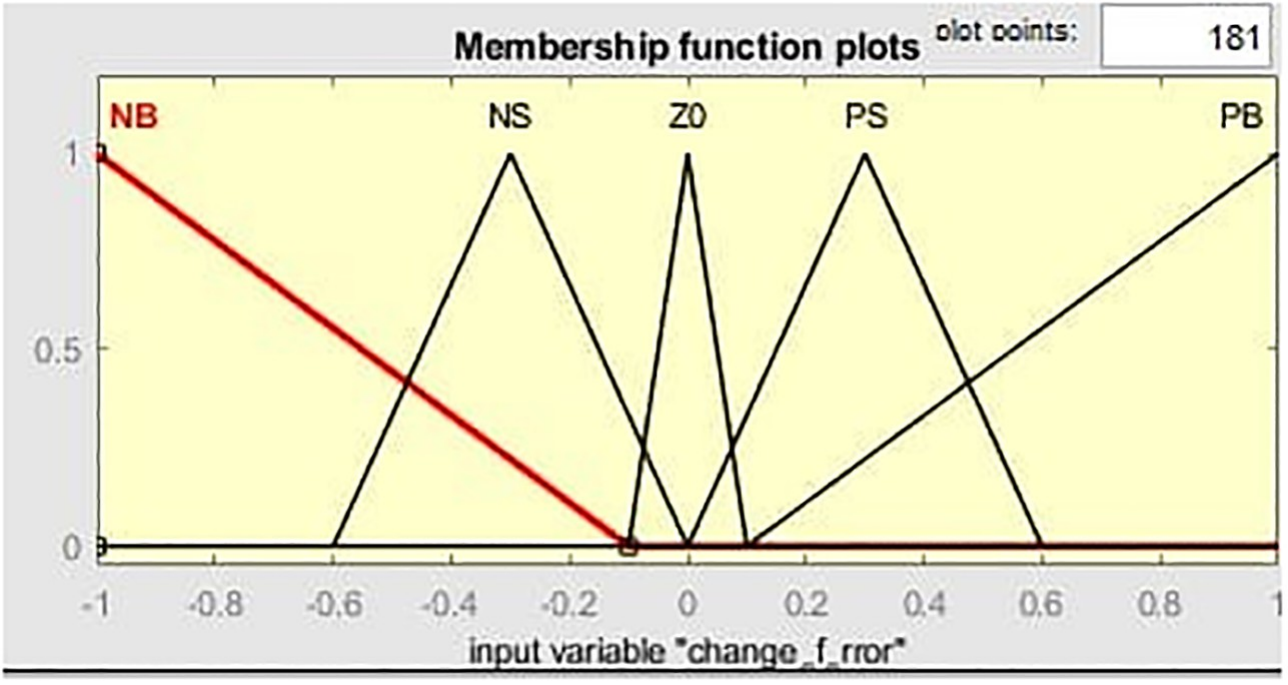
Membership function for change of error.

**Fig 13:**
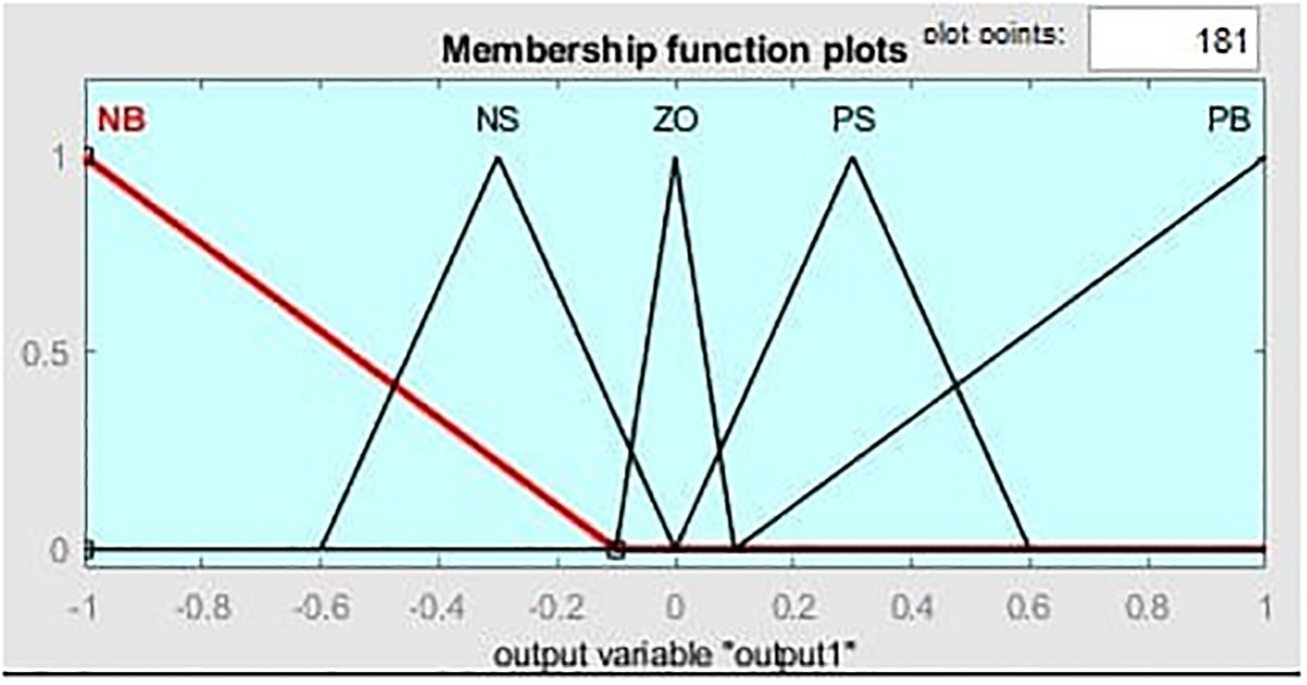
Membership function for the output variable x.

**Fig 14:**
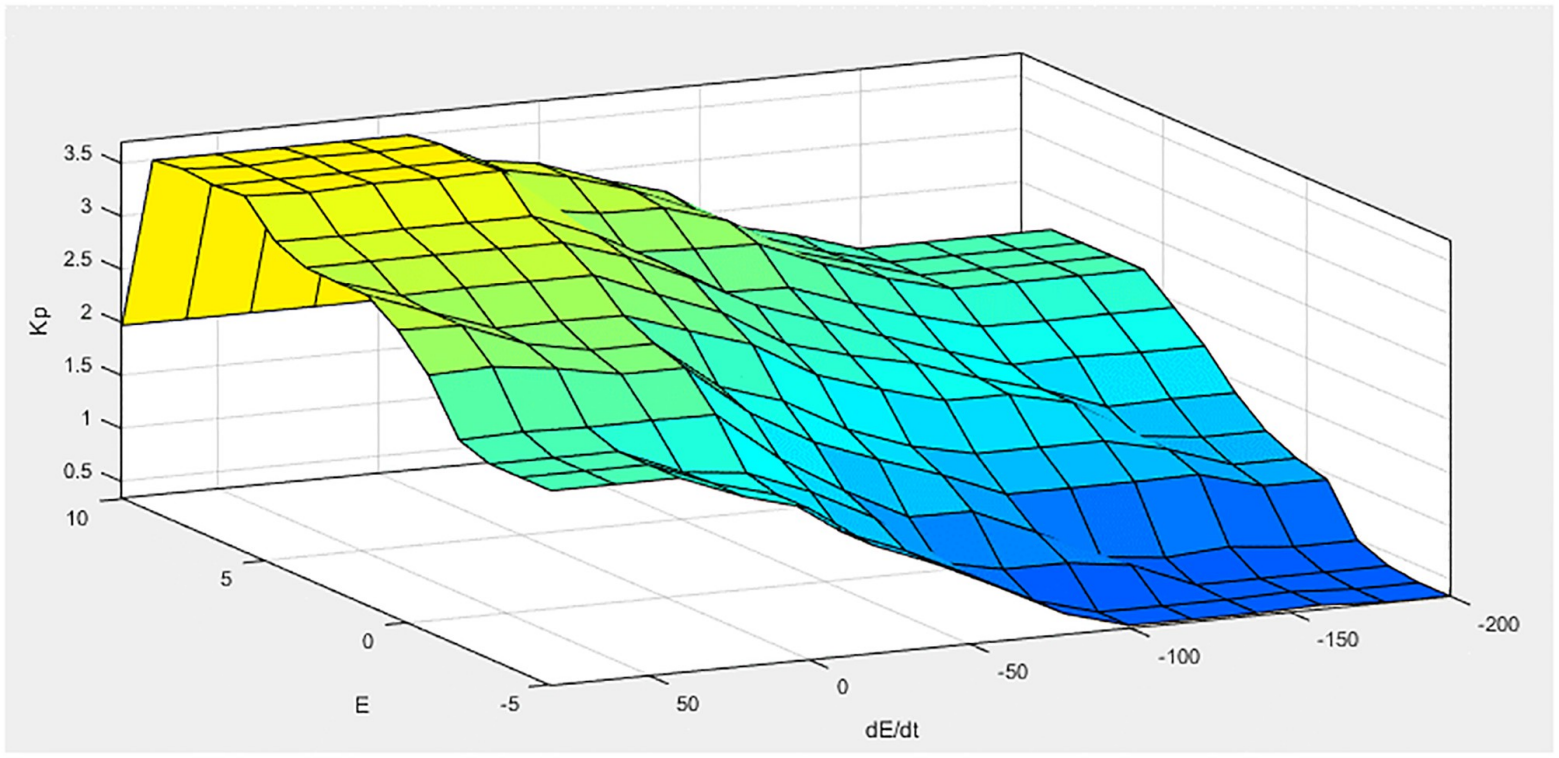
3-D surface view of rule base.

Fig 11 illustrates the MF for the input linguistic variable ‘error’ and both the input are distinct on the stabilized area of [-1 1]. While Fig 12 represent MF for change of error. MF for the output linguistic variable x is given in Fig 13. For these linguistic variables, five membership functions are chosen. Rule base: We developed rules for the two input linguistic variables ‘error’ and ‘change of error’ and the required output for the FLC using this MF. These two input are divided into five group’s i.e PS: Positive Small NS: Negative small, ZO: Zero, NB: Negative Big and PB Positive Big. These fuzzy control rule is shown Table 3 and 3-D surface view of rule base is given in Fig 14.

**Table 3:** Fuzzy rule base table.

e/e′	NB	NS	ZO	PS	PB
NB	NB	NB	NB	NS	ZO
NS	NB	NB	NS	ZO	PS
ZO	NB	NS	ZO	PS	PB
PS	NS	ZO	ZO	PS	PB
PB	ZO	PS	PB	PB	PB

### 0.2 Fuzzy inference system based controller for DC-DC boost converter

**Fig 15:**
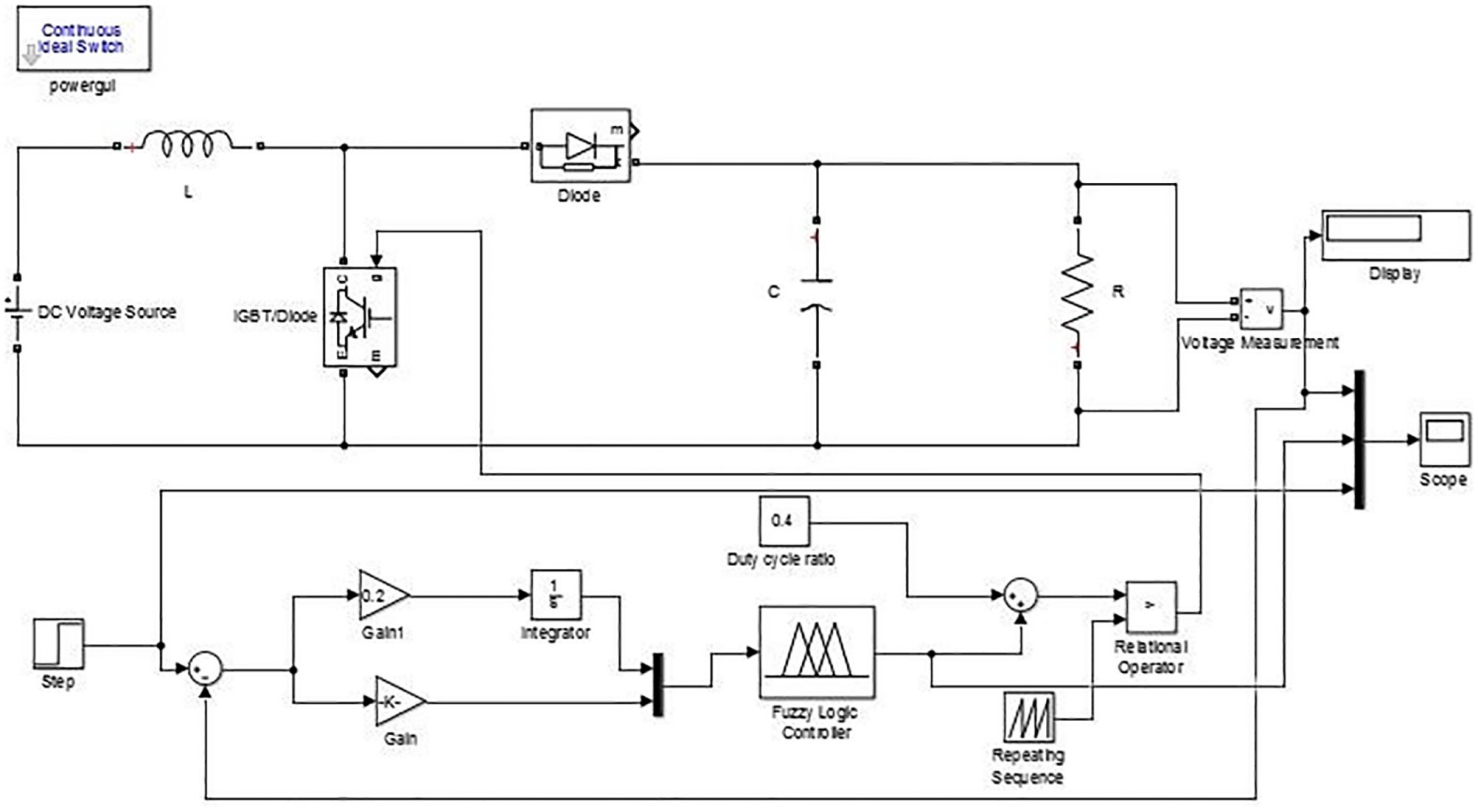
Simulink diagram of boost converter with FLC considering case-1, 2, and 3.

FIS based controller, has been design and implemented for DC-DC boost converter by employing the same parameters of the PID controller. The performance of the system has been assessed for three different cases depicted in Fig 15. Table 4 shows parameter for the boost converter with fuzzy implementation. The buck converter can benefit from the application of the following heuristic knowledge rules. 1) If the error is significantly different from zero, the duty cycle should change significantly. 2) If the error is close to zero, the duty cycle should change only slightly. 3) If the error is close to zero but the variation of the error is large, the duty cycle should be adjusted. In this way, overshoots can be avoided. 4) The duty cycle change does not need to be as significant as it would be if the error changed in such a way that the output approaches zero error, but the error is still significantly greater than zero. To incorporate this information, either modify the input MF or the outcomes of the rules (output singletons). Maintaining the controller’s input-output relationship is only possible in a small region of the control surface, close to the zero-error and zero-change points, assuming the membership functions and rules do not change. For inputs constrained to this range, the performance of the controller can be predicted using the same tiny signal models and linear control techniques as the original controller. There are two sources of data that feed into the FIS. The control error is the difference between the reference signal and the output signal, and the drift in the error is the rate at which the control error is increasing or decreasing.

**Table 4:** Parameters of the boost converter with FLC based controllers.

Parameters	Values	Parameters	Values
V_*in*_	100V	Inductor(L)	4mH
Capacitor (C)	100*μ*F	Resistor(R)	30 ohm
Frequency (f)	2KHz	Proportional (K_*p*_)	0.002
Integral (K_*i*_)	0.22	Derivative (K_*d*_)	10

**Fig 16:**
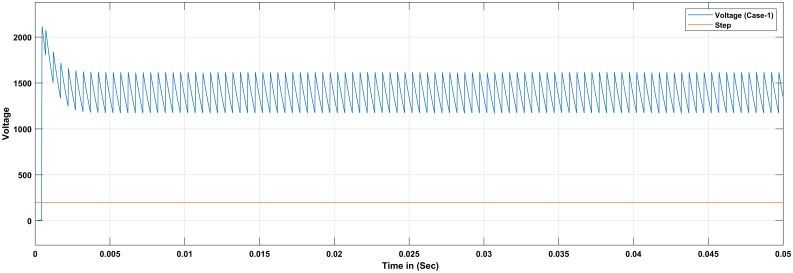
Results of boost converter with FLC considering case-1.

**Fig 17:**
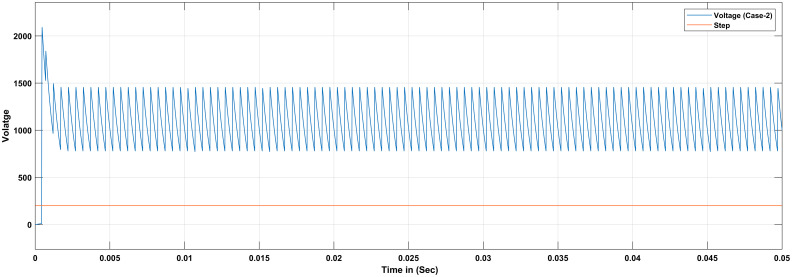
Results of boost converter with FLC considering case-2.

Different scenarios have also been validate using Fuzzy logic based PID controller. For case 1 the simulation was run for 0.05 seconds using Matlab/Simulink tool. Here in this case single 15 ohms resistor are used. The reference voltage increases from 200V to 300V. Fig 16 shows the simulation of Boost Converter with FLC at resistive load R = 15 ohms. The ripple constantly fluctuate between 1200V and 1500V even when the reference voltage increases from 200V to 300V. For case 2 the simulation was run for 0.05 seconds using Matlab/Simulink tool. Here in this case two resistor are connected in parallel while, each resistor have 15 ohms. So the total resistive load decreases from 15 ohms and the output response of the system is provided in Fig 17. For case 3 the simulation was run for 0.05 seconds using Matlab/Simulink tool at total resistive load 30 ohms. So the ripple increases and fluctuate between 800V and 1400V. In case-3 it can be conclude that the ripple become small as compared to first two cases and fluctuate between 1750V and 1950V.

**Fig 18:**
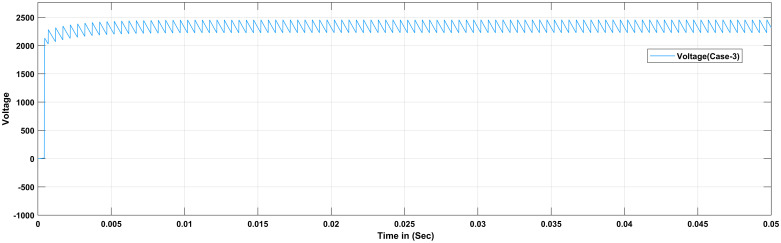
Results of boost converter with FLC considering case-3.

**Fig 19:**
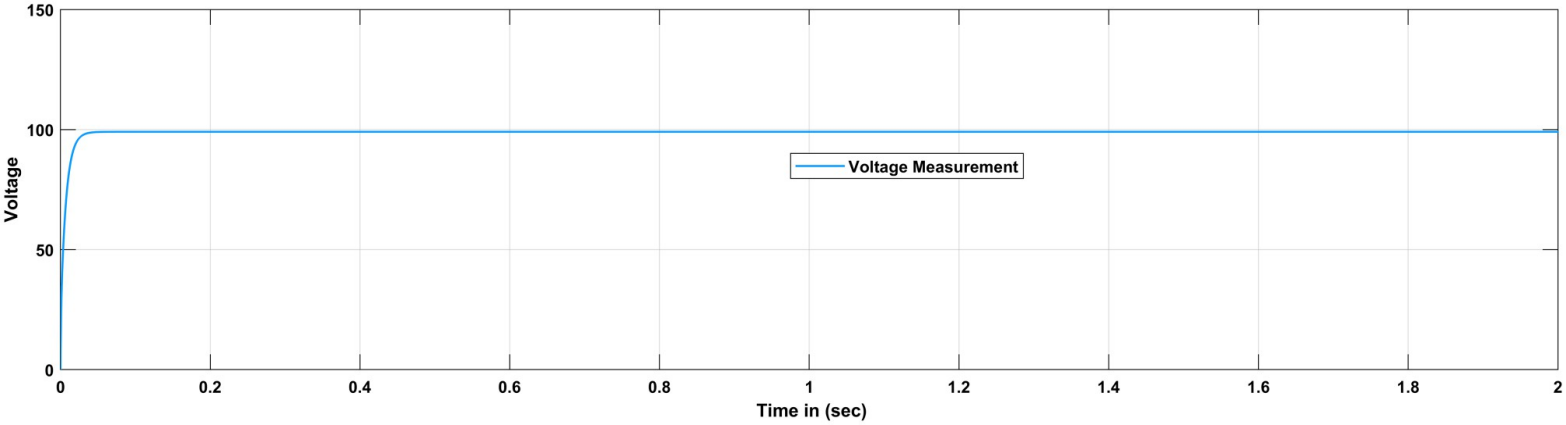
Results of boost converter with FLC considering no load.

FLC is used to simulate a DC-DC converter with the same parameters as those listed in Table 2. The result is shown in Fig 18. The error and derivative of error are fed into the FLC, and the output of the FLC is compared to a repeating sequence in order to generate a PWM signal that turns on and off the switch. From the Simulink model results different response of the outputs are analyzed in terms of overshoot for PI and fuzzy based controller. Finally, the results are compared in term of percentage improvement and perceived that the overshoot caused by change in the input voltage is 430 mV when the PID controller is employed. The overshoot is reduced to 320 mV when the proposed FLC is used, which is 74 percent less than when the PID controller is utilized. Aside from that, FLC has a short recovery time. Furthermore, it can be also perceived that the overshoot caused by the change in load is 210 mV when the PID controller is used. The overshoot is reduced to 180 mV when the proposed FLC is used, which is 86 percent less than when the proposed PID controller is used. Fig 19 represents the output response of the system with no load and the transient response parameters are obtained in Table 5. Fig 19 shows that that the proposed FLC based PID controller completely eliminate overshoot compare to PID controller and employed for the same system. Furthermore, it can be also observed from the Table 5 that our suggested fuzzy based controller perform excellent for no load in respect of transient parameters like rise time, settling time and overshoot as compared to PID controller.

**Table 5:** Comparison of PID and fuzzy PID controller for different cases.

Parameters	Rise time (Sec)	Overshoot (percentage)	Time settling (Sec)
PID (case-1)	0.023	12.14	-
PID (case-2)	0.045	13.08	-
PID (case-3)	0.048	14.90	-
PID (No-load)	0.016	0.000	0.02
Fuzzy PID (case-1)	0.00125	09.13	-
Fuzzy PID (case-2)	0.00175	10.09	-
Fuzzy PID (case-3)	0.00210	11.46	-
Fuzzy PID (No-load)	0.043	12.96	0.014
[31]	0.02	14.56	0.018

## Conclusion

In this paper, PID and FLC controllers are designed to control the output voltage of the inverter. The results of the output voltage of the inverter reveals that the FLC based controller has minimum overshoot and produces high gain in the output voltage compared to the PID controller. Moreover, five different reminiscence functions have been performed to observe the response of the system for the DC-DC boost converter using FIS, which gives a lower settling time for the FLC compared to the PID controller. Moreover, the fuzzy controller shows good stabilization characteristics while it does not need to know the mathematical model of the boost converter in the controller design process compared to the PID controller. Therefore, it can be concluded that the FLC provides better performance in terms of overshoot limitation and sensitivity to parameter changes compared to PID controller. The FLC also shows significant reduction in noise and settling time during switching operation compared to PID controller. Therefore, this study shows that the FLC is an intelligent controller with good transient response for the proposed problem compared to the PID controller. The experimental results will be considered in future studies to evaluate the performance of the same system as well as more complex system incorporating with fuel cell in distributed power system.

## Supporting information

S1 File (TXT)
